# Maternal and Perinatal Outcomes in Second Hemoglobin Measurement in Nonanemic Women at First Booking: Effect of Altitude of Residence in Peru

**DOI:** 10.5402/2012/368571

**Published:** 2012-04-19

**Authors:** Gustavo F. Gonzales, Vilma Tapia, Alfredo L. Fort

**Affiliations:** ^1^Department of Biological and Physiological Sciences, Universidad Peruana Cayetano Heredia, Lima 31, Peru; ^2^Instituto de Investigaciones de la Altura, Universidad Peruana Cayetano Heredia, Lima 31, Peru; ^3^Department of Reproductive Health and Research, World Health Organization, Geneve, Switzerland

## Abstract

*Objective*. To determine changes in hemoglobin concentration at second measurements after a normal hemoglobin concentration was detected at first booking during pregnancy at low and at high altitudes. *Methods*. This is a secondary analysis of a large database obtained from the Perinatal Information System in Peru which includes **379,816** pregnant women and their babies from 43 maternity units in Peru. *Results*. Most women remained with normal hemoglobin values at second measurement (75.1%). However, 21.4% of women became anemic at the second measurement. In all, 2.8% resulted with moderate/severe anemia and 3.5% with erythrocytosis (Hb>14.5 g/dL). In all cases Hb was higher as altitude increased. Risk for moderate/severe anemia increased associated with higher gestational age at second measurement of hemoglobin, BMI <19.9 kg/m^2^, living without partner, <5 antenatal care visits, first parity, multiparity, and preeclampsia. Lower risk for moderate/severe anemia was observed with normal high Hb level at first booking living at moderate and high altitude, and high BMI. *Conclusion*. Prevalence of anemia increases as pregnancy progress, and that a normal value at first booking may not be considered sufficient as Hb values should be observed throughout pregnancy. BMI was a risk for anemia in a second measurement.

## 1. Introduction

Maternal moderate and severe anemia have been considered as conditions that affect adversely maternal and perinatal outcomes. For such reason the identification of these cases is important since appropriate treatment may reduce the risk for adverse outcomes. Iron is the most common treatment currently used to control iron deficiency and anemia particularly in low-income countries, where anemia is more frequent [1].

Physiologically, hemoglobin concentration drops during gestation [2], and for such reason the hemoglobin cutoff to define anemia was set at 11 g/dL of Hb [1]. Maternal Hb levels are higher at high altitudes in all trimesters of pregnancy than at sea level [3]. However, the reduction of hemoglobin levels during pregnancy occurs in populations at low and at high altitudes [3].

This is due to plasma volume expansion as a mechanism to improve arterial uterine flow to the placentae [4]. High arterial uterine blood flow is associated with better birthweight [5].

Several studies have shown that prevalence of anemia increases from first to third trimester [6]. Thus, a normal Hb value at first booking in the first trimester does not preclude the presence of anemia in a second measurement of hemoglobin as pregnancy advances. A recent study showed a higher risk for anemia in pregnant women with low body mass index (BMI) [7]. Underweight is a common condition in developing countries [8]. Since, anemia, particularly moderate and severe anemia is associated with high risk for low birthweight [3, 9, 10], it is important to monitor hemoglobin values during pregnancy.

The present study has been designed to determine changes in hemoglobin concentration at second measurements after a normal hemoglobin concentration was detected at first booking during pregnancy at low and at high altitudes. We will determine rates of anemia, normal hemoglobin values and erythrocytosis (Hb >14.5 g/dL). In addition, risk factors for moderate/severe anemia will be assessed in a multivariable analysis.

## 2. Methods

This is a secondary analysis of a large data base obtained from the Perinatal Information System in Peru which includes **379,816** pregnant women and their babies obtained from 43 Maternity units in Peru. Data were obtained at the three geographical regions in Peru: coast, highlands, and Amazon. The study was authorized by the Institutional Review Board at the Universidad Peruana Cayetano Heredia. As the study used secondary analysis of data, the study was exempt from formal review.

From the total sample size, a selection was made of all women diagnosed as nonanemic and without erythrocytosis at first booking (Hb ranging from 11 to 14.5 g/dL) and having a second measurement of hemoglobin during the same pregnancy. This subsample includes 89,294 pregnant women. From these, 57,231 pregnant women were living at low altitude (<2000 m), 11,349 women at moderate altitude (2000–3000 m), and 20,714 women were living at high altitudes (>3000 m).

Data corresponds to pregnancies that occurred between 2000 and 2010. Data on gestational age at first and second hemoglobin measurement were recorded. With a second hemoglobin value, rates of pregnant women with anemia (Hb < 11 g/dL), normal hemoglobin values (11–14.5 g/dL), and erythrocytosis (Hb>14.5 g/dL) were determined.

As demonstrated previously, no correction of hemoglobin cutoff to define anemia at high altitude is needed [3]; hence, the present study used the definition of anemia as suggested WHO for populations at sea level [1, 11]. The degrees of anemia were classified as mild (Hb 9.0–10.9 g/dL), moderate (Hb 7.0–< 9 g/dL), and severe (Hb < 7 g/dL).

Body mass index was calculated from prepregnancy body weight and height. Data were self-reported. Low BMI was defined as <19.9 Kg/m^2^ and high BMI as >25 Kg/m^2^.

Altitude was defined as low (0–1999 m), moderate (2000–2999 m), and high (≥ 3000 m) [10].

The following perinatal outcomes were assessed: stillbirths, preterm deliveries and small for gestational age. Stillbirths were diagnosed when fetal deaths occurred after 20 weeks of gestation or with a weight higher than 500 g. Preterm births were defined as deliveries before 37 weeks of gestational age. Small for gestational age was defined if birthweight was below the 10th percentile of the Williams reference chart [12].

The adverse maternal outcomes assessed were preeclampsia, premature rupture of membranes (PROM), and postpartum hemorrhage (PPH). Preeclampsia was defined as the presence of pregnancy-induced hypertension (systolic pressure of ≥140 mm Hg and/or diastolic pressure of ≥90 mm Hg) and proteinuria (≥300 mg/24 h) after 20 weeks of gestation. PROM was defined as rupture of fetal membranes previous to the onset of labor. PPH was defined as women after parturition with blood losses of 500 mL or more.

Data were assessed using the STATA program (Stata ver. 10) (Stata, College Station, TX). Data are assessed according level of altitude (low, moderate, and high) and according to degree of anemia (moderate/severe and mild), and erythrocytosis. Quantitative data were expressed as mean ± standard deviation (SD). Homogeneity of variance was performed with the Breslow-Day test. If data were normally distributed, then a one-way analysis of variance were used (ANOVA). Differences between pair of means were assessed by the Scheffe's test. Data expressed in proportions were assessed by the Chi-square test. The Confidence interval (CI) was defined at 95% for each variable. The prevalences of women with moderate/severe anemia (Hb <9 g/dL), mild anemia (Hb 9–10.9 g/dL), normal hemoglobin (Hb 11–14.5 g/dL), and erythrocytosis (Hb >14.5 g/dL) were calculated for the total sample, as well as for groups at different altitudes (low, moderate, and high altitude). Adverse perinatal and maternal outcomes were assessed according level of hemoglobin at second measurement. Estimates of crudes and adjusted odds ratio (OR) with 95% CI were computed as measures of association between the variables. Adjusted ORs were derived through logistic regression models. A logistic regression analysis to determine the risk for moderate/severe anemia at second hemoglobin measured was assessed. The estimates were adjusted for gestational age at second hemoglobin, hemoglobin value at first measurement, altitude, maternal age, body mass index, maternal education, marital status, prenatal care visits, parity, the presence of preeclampsia, cardiopathy, and diabetes. Significance was defined at a value of *P* < 0.05 for all statistical analysis.

## 3. Results

Concentration of hemoglobin at first booking in nonanemic women increased as altitude increased (*P* < 0.05), whereas birthweight decreased as altitude increased. No differences were observed in gestational age at birth at the three altitude groups studied. Concentration of hemoglobin at second measurement also was increased as altitude increases, but values were lower than those at first measurement, particularly at low altitude, in which values were reduced to 94%, whereas at moderate altitude. They were reduced to 98.4% and at high altitude to 99.2%. Gestational age at first and second hemoglobin was higher as altitude of residence increased (Table 1).

**Figure 1:**
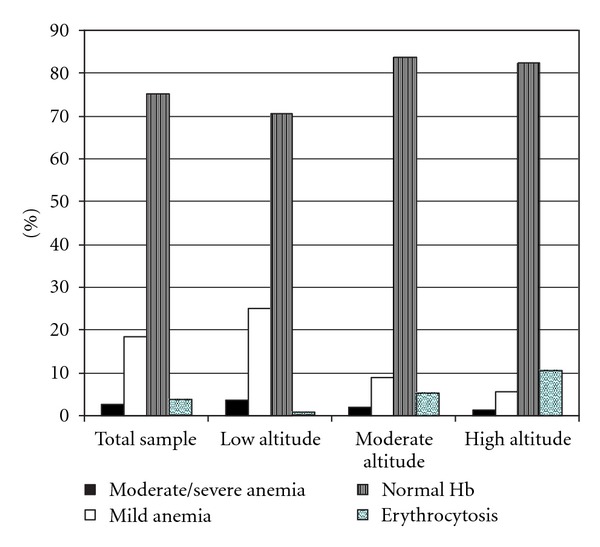
Rates of moderate/severe anemia, mild anemia, normal hemoglobin values (Hb: 11–14.5 g/dL), and erythrocytosis (Hb>14.5 g/dL) in the second hemoglobin measurements in pregnant women diagnosed as nonanemic at first booking in Peru.

Most of the studied sample remained with normal hemoglobin values at second hemoglobin measurement (75.1). However, 21.4% of women became anemic at the second Hb measurement. In all, 2.8% resulted with moderate/severe anemia and 3.5% with erythrocytosis (Figure 1).

Women with anemia in the second Hb measurement showed lower Hb measurements in the first Hb. Conversely, women with erythrocytosis at the second Hb measurement showed higher Hb levels in the first measurement (Table 2). In all cases Hb was higher as altitude increased.

The rate of stillbirths was higher with moderate/severe anemia and with erythrocytosis (*P* < 0.05), which remained higher after controlling for confounders (Table 3).

The rate of preterm births was increased in women with severe anemia (9.01%; OR = 1.81). After adjusting for confounders, moderate/severe anemia (OR = 1.73) and mild anemia (OR = 1.28) were risks for preterm deliveries (Table 4).

The rate of SGA was significantly higher in mothers with erythrocytosis, almost twice, than in women with normal hemoglobin. Lowest rate of SGA was observed with a maternal Hb level of 9–10.9 g/dL (mild anemia) (Table 5).

The rate of preeclampsia was significantly higher in moderate/severe and mild anemia and in mothers with erythrocytosis, which remained after controlling for confounders (Table 6). Erythrocytosis reduced risk for PROM (Table 7).

PPH increased significantly with mild and moderate/severe anemia. The highest risk for PPH was observed with moderate/severe anemia (twenty times higher than in woman with normal Hb levels) (Table 8).

An increased risk for moderate/severe anemia was associated with higher gestational age at second measurement of hemoglobin, BMI < 19.9 kg/m^2^, living without partner, prenatal visits care <5, first parity, multiparity, and preeclampsia.

Lower risk for moderate/severe anemia were observed with normal high Hb level at first booking, living at moderate and high altitude, and with high BMI (Table 9).

## 4. Discussion

The present study has been designed to determine changes in Hb levels after a first Hb measurement in 89,294 pregnant women detected normality (Hb 11–14.5 g/dL). According to the study results 21.4% of these women become anemic. An increase in the rate of anemia according to pregnancy progress has been previously published in other settlings [6]. Moderate/severe anemia has been characterized as the conditions more associated with maternal and perinatal adverse outcomes. Our results demonstrated that 2.8% of women classified as nonanemic in the first Hb measurement showed moderate/severe anemia in the second measurement. Thus, it is important to monitor Hb levels at least twice during pregnancy. Our data showed that moderate/severe anemia were associated with stillbirths, preterm births, preeclampsia, and PPH.

The strength of this study is that it was done thanks to a large sample of women recorded through a Perinatal Information System that included **379,816** pregnant women and their newborn babies from different maternity units throughout Peru. This database is an important tool for monitoring conditions and trends related to pregnancies and newborns, including service characteristics and women's risk factors.

Data revealed that a percentage of women became anemic during the progression of pregnancy despite having a first normal Hb measurement. This is an important finding since moderate and severe anemia are harmful for mothers and neonates [13]. Risks for moderate/severe anemia are preventable, or can be managed through appropriate management, such as with prenatal care, parity, preeclampsia, and low BMI. Increasing the number of prenatal visits will result in better monitoring of hemoglobin values, and can control or reduce the effect of preeclampsia. Low BMI is a characteristic of low-income countries and is associated with increased risk of maternal anemia, low birthweight, and preterm births [7]. The present results are in accordance with other studies where low prevalence of anemia was observed in women with highest BMI, and high prevalence of moderate/severe anemia were associated with malnutrition [14, 15].

Our data suggest that in situation in which a normal hemoglobin value was first observed in a woman with low body mass index, supplementation with iron should be recommended.

It is interesting to note that populations living at moderate and high altitudes had lower risk for moderate/severe anemia in a second measurement of hemoglobin during pregnancy. This suggests that women living in these altitudes bearing normal hemoglobin values at first booking should not be treated with iron supplementation. In recent years, it has been demonstrated that anemia defined as less than 11 g/dL of Hb is lower at moderate and at high altitudes, since populations at these altitudes are characterized by increased hemoglobin values to compensate for the effect of altitudinal hypoxia [3, 10].

### 4.1. Limitations

The database does not provide information about the etiology of anemia. However, we assumed that most cases of anemia were related to iron deficiency, which was supported by the finding that low BMI was associated with moderate/severe anemia. Prepregnancy weight was measured by self-reported interviews. This may be affected by recall bias. However, results of our analysis were similar to those obtained in a study in which BMI was obtained at first prenatal care [7]. The absence of daily food intake information was another limitation of the study. In addition, data on iron supplementation was not recorded in the SIP. It is possible that erythrocytosis found in the second measurement of Hb after a normal value of Hb at first booking could be due to the effect of iron supplementation, since in Peru a Ministry of Health regulation orders pregnant women be supplemented with iron. However, low adherence may explain the low prevalence of erythrocytosis in a second Hb measurement during pregnancy [16].

## 5. Conclusions

The results of this study confirm that prevalence of anemia increases as pregnancy progresses and that a normal value at first booking should not be considered sufficient, as further hb values should be indicated and observed during the course of pregnancy. low BMI was an important risk for anemia in a second measurement.

## Figures and Tables

**Table 1:** Concentration of maternal hemoglobin (Hb) at first and second booking, gestational age (GA) at first and second hemoglobin measurement, gestational age at delivery and birthweight in populations at low, and moderate and high altitude in Peru.

Altitude	1st Hb (g/dL)		2nd Hb (g/dL)		GA 1st Hb (weeks)		GA 2nd Hb (weeks)		Gestational age (weeks)		Birthweight (g)	
	X ± SD		X ± SD		X ± SD		X ± SD		X ± SD		X ± SD	
Low	12.1	0.80	11.4	1.34	17.2	8.2	36	11.6	38.9	1.76	3262	512
Moderate	12.7	0.90	12.5	1.43	19.4	8.36	36.7	4.6	38.8	2.07	3123	529
High	13.0	0.88	12.9	1.40	20.6	8.3	37.3	3.9	38.8	1.89	3095	489
*P*	<0.05		<0.05		<0.05		<0.05				<0.05	
Total	12.4	0.91	11.9	1.52	18.3	8.4	36	4.5	38.9	1.83	3206	515

g/dL = grams per deciliter; *P* = Probability. One-way ANOVA. *P* < 0.05 for each column except gestational age (*P* > 0.05).

**Table 2:** Maternal hemoglobin values at first booking in women diagnosed as anemic (moderate/severe and mild), normal or erythrocytic at second hemoglobin measurement*.

Altitude (sample)	Maternal hemoglobin (g/dL) at first booking							
	Moderate/severe*		Mild*		Normal (Hb: 11–14.5 g/dL)*		Hb > 14.5 g/dL*	
	X ± SD		X ± SD		X ± SD		X ± SD	
Total (89,294)	12.10	0.84	12.10	0.82	12.50	0.91	13.19	0.86
Low (57,231)	11.99	0.77	12.02	0.77	12.22	0.81	12.51	0.87
Moderate (11,349)	12.55	0.87	12.47	0.93	12.74	0.89	13.06	0.86
High (20,714)	12.66	0.94	12.70	0.94	13.03	0.87	13.33	0.81

ANOVA one way. *P* < 0.05 except moderate/severe anaemia: moderate altitude versus high altitude *P* = 0.293.

**Table 3:** Crude and adjusted models to evaluate the association between the value of the second measurement of maternal hemoglobin (Hb) and stillbirths in women with normal hemoglobin levels (11–14.5 g/dL) at first booking in Peru.

Maternal Hb (g/dL)	Sample *N*	Stillbirths (*n* = 846)					
		*n*	%	Crude OR	Adjusted OR	CI at 95%	
<9	2,575	68	2.64	3.06*	2.76*	2.11	3.62
9–10.9	16,576	140	0.84	0.96	1.02	0.84	1.25
11–14.5	67,022	587	0.88	1.0	1.0		
>14.5	3,121	51	1.63	1.88*	1.48*	1.09	2.04

*Crude OR *P* < 0.05. Model adjusted for gestational age at first Hb, maternal age, altitude, maternal education, marital status, body mass index, antenatal care, parity, gestational diabetes, and cardiopathy (current pregnancy).

**Table 4:** Crude and adjusted models to evaluate the association between the value of the second measurement of maternal hemoglobin (Hb) and preterm births in women with normal hemoglobin levels (11–14.5 g/dL) at first booking in Peru.

Maternal Hb (g/dL)	Sample *N*	Preterm Births (*n* = 4,932)					
		*n*	%	Crude OR	Adjusted OR	CI at 95%	
<9	2,575	232	9.01	1.81*	1.73*	1.49	2.00
9–10.9	16,576	1,043	6.29	1.23*	1.28*	1.19	1.38
11–14.5	67,022	3,468	5.17	1.0	1.0		
>14.5	3,121	189	6.06	1.18*	1.02	0.87	1.20

*OR *P* < 0.05. Model adjusted for gestational age at first Hb, maternal age, altitude, maternal education, marital status, body mass index, antenatal care, parity, preeclampsia, gestational diabetes and cardiopathy, and infection of urinary tract (current pregnancy).

**Table 5:** Crude and adjusted models to evaluate the association between the value of the second measurement of maternal hemoglobin (Hb) and small for gestational age in women with normal hemoglobin levels (11–14.5 g/dL) at first booking in Peru.

Maternal Hb (g/dL)	Sample *N*	SGA (*n* = 9,239)					
		*n*	%	Crude OR	Adjusted OR	CI at 95%	
<9	2,575	243	9.44	0.88	0.87*	0.76	1.00
9–10.9	16,576	1,333	8.04	0.74*	0.82*	0.77	0.88
11–14.5	67,022	7,040	10.5	1.0	1.0		
>14.5	3,121	623	19.9	2.12*	1.66*	1.51	1.83

*OR *P* < 0.05. Model adjusted for gestational age at first Hb, maternal age, altitude, maternal education, marital status, body mass index, antenatal care, parity, preeclampsia, gestational diabetes and cardiopathy, (current pregnancy), and history of low birthweight.

**Table 6:** Crude and adjusted models to evaluate the association between the value of second measurement of maternal hemoglobin (Hb) and preeclampsia in women with normal hemoglobin levels (11–14.5 g/dL) at first booking in Peru.

Maternal Hb (g/dL)	Sample *N*	Preeclampsia (*n* = 5, 385)					
		*n*	%	Crude OR	Adjusted OR	CI at 95%	
<9	2,575	283	10.9	2.02*	1.92*	1.69	2.19
9–10.9	16,576	1,041	6.28	1.09*	1.06	0.99	1.14
11–14.5	67,022	3,851	5.75	1.0	1.0		
>14.5	3,121	210	6.73	1.18*	1.32*	1.14	1.53

*OR *P* < 0.05. Model adjusted for gestational age at first Hb, maternal age, altitude, maternal education, marital status, body mass index, antenatal care, parity, gestational diabetes and cardiopathy, infection of urinary tract (current pregnancy), and history of preeclampsia.

**Table 7:** Crude and adjusted models to evaluate the association between the value of the second measurement of maternal hemoglobin (Hb) and premature rupture of membranes (PROM) in women with normal hemoglobin levels (11–14.5 g/dL) at first booking in Peru.

Maternal Hb (g/dL)	Sample *N*	PROM (*n* = 5,821)					
		*n*	%	Crude OR	Adjusted OR	CI at 95%	
<9	2,575	158	6.14	0.90	0.92	0.78	1.09
9–10.9	16,576	999	6.03	0.89*	0.93	0.86	1.00
11–14.5	67,022	4,498	6.71	1.0	1.0		
>14.5	3,121	166	5.32	0.78*	0.71*	0.60	0.84

*OR *P* < 0.05. Model adjusted for gestational age at first Hb, maternal age, altitude, maternal education, marital status, body mass index, antenatal care, parity, preeclampsia, gestational diabetes, cardiopathy, and infection of urinary tract (current pregnancy).

**Table 8:** Crude and adjusted models to evaluate the association between the value of the second measurement of maternal hemoglobin (Hb) and postpartum hemorrhage (PPH) in women with normal hemoglobin levels (11–14.5 g/dL) at first booking in Peru.

Maternal Hb(g/dL)	Sample *N*	PPH (*n* = 728)					
		*n*	%	Crude OR	Adjusted OR	CI at 95%	
<9	2,575	219	8.50	19.3*	20.4*	16.9	24.5
9–10.9	16,576	175	1.06	2.21*	2.59*	2.14	3.14
11–14.5	67,022	321	0.48	1.0	1.0		
>14.5	3,121	13	0.42	0.86	0.73	0.41	1.27

*OR *P* < 0.05. Model adjusted for gestational age at first Hb, maternal age, altitude, maternal education, marital status, body mass index, antenatal care, parity, preeclampsia, gestational diabetes and cardiopathy (current pregnancy).

**Table 9:** Risk for moderate/severe anemia at second measurement in women with normal hemoglobin (Hb) levels (11–14.5 g/dL) at first booking in Peru.

Risk for moderate/severe anemia	Odds ratio	S.E	*Z*	*P* > *Z*	95% CI
Gestational age at 2nd Hb Hb at first booking Moderate altitude High altitude Age <20 years Age >34 years BMI <19.9 kg/m^2^ BMI >25.0 Kg/m^2^ Low education level Without partner Prenatal care <5 First parity Parity ≥3 Preeclampsia Cardiopathy Gestational diabetes	1.05* 0.74 0.57 0.40 1.07 0.97 1.19* 0.84 1.01 1.16* 1.13* 1.14* 1.25* 1.96* 2.09 1.80	0.006 0.019 0.041 0.028 0.056 0.074 0.071 0.041 0.054 0.067 0.047 0.056 0.117 0.128 0.891 0.832	9.21 −11.60 −7.62 −12.92 1.24 −0.43 2.87 −3.52 0.13 2.64 2.98 2.60 2.38 10.28 1.74 1.26	0.000 0.000 0.000 0.000 0.216 0.668 0.004 0.000 0.900 0.008 0.003 0.009 0.017 0.000 0.083 0.207	1.04–1.07 0.70–0.78 0.49–0.66 0.35–0.46 0.96–1.18 0.83–1.12 1.06–1.33 0.76–0.93 0.91–1.12 1.04–1.30 1.04–1.23 1.03–1.25 1.04–1.50 1.72–2.23 0.91–4.82 0.72–4.45

Number of observations: 89,294 mothers. *Significant at 5% level.

## References

[1] United Nations Children's Fund (2001). Iron deficiency anaemia assessment, prevention, and control: a guide for programme managers. *WHO/NHD/*.

[2] Roberfroid D, Huybregts L, Habicht J-P (2011). Randomized controlled trial of 2 prenatal iron supplements: is there a dose-response relation with maternal hemoglobin?. *American Journal of Clinical Nutrition*.

[3] Gonzales GF, Steenland K, Tapia V (2009). Maternal hemoglobin level and fetal outcome at low and high altitudes. *American Journal of Physiology*.

[4] Steer PJ (2000). Maternal hemoglobin concentration and birth weight. *American Journal of Clinical Nutrition*.

[5] Julian CG, Wilson MJ, Lopez M (2009). Augmented uterine artery blood flow and oxygen delivery protect Andeans from altitude-associated reductions in fetal growth. *American Journal of Physiology*.

[6] Baraka MA, Steurbaut S, Laubach M, Coomans D, Dupont AG Iron status, iron supplementation and anemia in pregnancy: ethnic differences.

[7] Liabsuetrakul T (2011). Is international or asian criteria-based body mass index associated with maternal anaemia, low birthweight, and preterm births among thai population?-An observational study. *Journal of Health, Population and Nutrition*.

[8] Adam I, Babiker S, Mohmmed AA, Salih MM, Prins MH, Zaki ZM (2008). Low body mass index, anaemia and poor perinatal outcome in a Rural Hospital in Eastern Sudan. *Journal of Tropical Pediatrics*.

[9] Bodeau-Livinec F, Briand V, Berger J (2011). Maternal anemia in Benin: prevalence, risk factors, and association with low birth weight. *American Journal of Tropical Medicine and Hygiene*.

[10] Gonzales GF, Tapia V, Gasco M, Carrillo CE Maternal hemoglobin concentration and adverse pregnancy outcomes at low and moderate altitudes in Peru. *Journal of Maternal-Fetal and Neonatal Medicine*.

[11] World Health Organization (2006). Prevalence of anemia. *Reproductive Health Indicators. Guidelines for Their Generation, Interpretation and Analysis for Global Monitoring*.

[12] Williams RL, Creasy RK, Cunningham GC (1982). Fetal growth and perinatal viability in California. *Obstetrics and Gynecology*.

[13] Rohilla M, Raveendran A, Dhaliwal LK, Chopra S (2010). Severe anaemia in pregnancy: a tertiary hospital experience from northern India. *Journal of Obstetrics and Gynaecology*.

[14] Bondevik GT, Ulstein M, Lie RT, Rana G, Kvåle G (2000). The prevalence of anemia in pregnant Nepali women a study in Kathmandu. *Acta Obstetricia et Gynecologica Scandinavica*.

[15] Sharma V, Sharma A (1992). Health profile of pregnant adolescents among selected tribal populations in Rajasthan, India. *Journal of Adolescent Health*.

[16] Munayco C, Arias L, Gambirazio C, Suarez L Adherencia a la suplementación con hierro durante la gestación en las Direcciones de Salud de Apurimac y Ayacucho.

